# Computational and Experimental Approaches to Reveal the Effects of Single Nucleotide Polymorphisms with Respect to Disease Diagnostics

**DOI:** 10.3390/ijms15069670

**Published:** 2014-05-30

**Authors:** Tugba G. Kucukkal, Ye Yang, Susan C. Chapman, Weiguo Cao, Emil Alexov

**Affiliations:** 1 Department of Physics, Clemson University, Clemson, SC 29634, USA; E-Mail: tugbak@clemson.edu; 2 Department of Genetics and Biochemistry, Clemson University, 049 Life Sciences Facility, 190 Collins Street, Clemson, SC 29634, USA; E-Mail: yyang9@clemson.edu; 3 Department of Biological Sciences, Clemson University, Clemson, SC 29634, USA

**Keywords:** single nucleotide polymorphism (SNP), pathogenic mutation, missense mutations, disease diagnostics, protein stability, protein interactions

## Abstract

DNA mutations are the cause of many human diseases and they are the reason for natural differences among individuals by affecting the structure, function, interactions, and other properties of DNA and expressed proteins. The ability to predict whether a given mutation is disease-causing or harmless is of great importance for the early detection of patients with a high risk of developing a particular disease and would pave the way for personalized medicine and diagnostics. Here we review existing methods and techniques to study and predict the effects of DNA mutations from three different perspectives: *in silico*, *in vitro* and *in vivo*. It is emphasized that the problem is complicated and successful detection of a pathogenic mutation frequently requires a combination of several methods and a knowledge of the biological phenomena associated with the corresponding macromolecules.

## 1. Introduction

There has been a rapid development of genome-wide techniques in the last decade along with significant lowering of the cost of gene sequencing, which generated rich and widely available genomic data. However, the interpretation of such genomic data as well as predicting the association of genetic differences with diseases still needs significant improvement. The problem stems from the fact that the effects of genetic differences on protein function vary widely making it difficult to decipher genotype-phenotype relationships. One plausible approach to reduce the ambiguity of disease association is to consider all molecular effects simultaneously by the means of combined efforts of *in silico*, *in vitro* and *in vivo* approaches. In this review, we summarize these computational and experimental methods that are used to reveal the potential impacts of genetic differences. The first section is devoted to the *in silico* methods and applications followed by a section on approaches and methods for *in vitro* investigations, and then a section reviewing the techniques and methods for *in vivo* studies. Finally, several methods described are applied to a case study to reveal the molecular mechanisms of several disease-causing mutations in two specific genes involved in X-linked mental retardations. These two genes, *MECP2* (coding for methyl CpG binding protein 2 (MeCP2), which is important for the normal function of the cell) and the *KDM5C* (coding for lysine (K)-specific demethylase 5C, which participates in transcriptional repression of neuronal genes) were selected based on our ongoing research.

## 2. *In Silico* Analysis of Pathogenic Mutations

**Figure 1:**
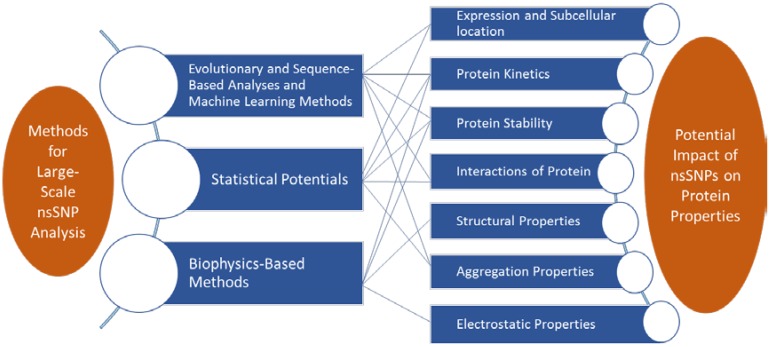
Flowchart illustrating the methods (**left**) used for assessment of potential impacts (**right**) of DNA mutations on protein properties and interactions. nsSNP, non-synonymous Single Nucleotide Polymorphism.

Genetic differences can cause a range of changes in the biophysical characteristics of macromolecules (DNA, RNA and proteins) including changes in stability, electrostatic properties, protein–protein, protein–DNA, protein–RNA and protein–membrane interactions, aggregation properties and structural characteristics. The latter includes changes in the H-bond network, pKa, hydrophobicity, flexibility and structural disorder. The potential impacts of mutations and methods used to study them are summarized in Figure 1. Understanding the rules that govern the changes caused by genetic mutations is crucial for disease diagnostics. Of particular interest are genetic differences resulting in amino acid changes of the corresponding protein, termed as non-synonymous single nucleotide polymorphism (nsSNP) or rare missense mutations. The methods developed to study the impact of single amino acid substitution cover a wide range of ideas from evolutionary and sequence-based predictions to detailed atomic energy-based methods, and several reviews albeit less comprehensive were published [1,2,3,4,5,6,7,8]. Here, we review these methods under broad categories and also provide examples of outcomes that advance our understanding of the changes in protein biophysical characteristics and interactions caused by mutations.

### 2.1. Sequence and Evolutionary Analyses and Machine Learning Methods

Disease-causing missense mutations are often found to occur at evolutionarily conserved positions that have a crucial role for protein structure and function. These sites are characterized through multiple sequence alignments, either with consensus sequences of the same protein in multiple organisms or with all homologues of the same protein. In many cases, the sequence identity implies structural similarity [9,10]. There have been a number of sequence-based methods developed to identify whether a missense mutation is pathogenic or not [11,12,13,14]. Basically, the results from the alignment are normalized and then the degree of tolerance for specific mutations is produced based on sequence conservation data. The biggest advantage of these methods is that large databases of mutations can be screened efficiently. However, these methods are very sensitive to multiple sequence alignments, therefore, different predictions may be produced depending on the depth of the alignment. The list of web servers based on these principles include Sorting Intolerant from Tolerant (SIFT) [12], Alignment Grantham-Variation, Grantham-Deviation (Align-GVGD) [15], Mutation Assessor [16] and Multivariate Analysis of Protein Polymorphism (MAPP) [17].

In addition, a number of methods combine evolutionary sequence conservation with other structural implications to characterize whether a mutation is pathogenic or not. These and other methods that incorporate different approaches concurrently are termed as combined methods. For example, PolyPhen-2 [18], makes the predictions based on sequence conservation, physico-chemical characteristics of the amino acids involved in the substitution along with the sequence environment of the mutation site and the structural features affected by the mutation. Other tools based on similar approaches include Mutation Taster [19], LS–SNP/PDB [20], SNPeffect [13], Predicting Protein Mutant Stability Change (MuStab) [21,22], MUpro [23], MutPred [24], SNPdbe [25], NetDiseaseSNP [26], HOPE [27] and SNPs3D [28].

In addition to, or in combination with evolutionary and sequence-based methods, several machine learning approaches such as Neural Networks, decision trees and Random Forests, Hidden Markov Models, Conditional Random Fields and Support Vector Machines (SVMs) have been used to predict the effects of single point mutations and specifically the stability changes upon mutations [29]. The key idea in machine learning is to direct the computer to learn how to solve a problem rather than providing the way to the solution.

Neural network and SVM methods have widely been applied in nsSNP analyses. Although neural network based methods can achieve comparable performance to SVMs, the latter has been more popular recently possibly due to the availability of general high-quality implementation of SVMs [30]. The I-Mutant [31] and I-Mutant2.0 [32] webservers use a neural network-based method and SVM, respectively, to predict the sign of free energy change upon mutations. When the free energy difference is obtained by subtracting the wild-type free energy from that of the mutant, the positive sign of the resulting free energy change indicates destabilization of the protein. I-Mutant, which uses structural information as well as temperature and pH to build the neurons, achieved an accuracy of 0.81 in predicting the sign of the free energy change upon mutations. I-Mutant was shown to outperform three other tools, namely the FoldX [33,34], Distance-Scaled, Finite Ideal Gas Reference (DFIRE) [35,36] and Prediction of Protein Mutant Stability Changes (PoPMuSiC) [37,38] servers, which use biophysics-based or statistical potentials. On the other hand, I-Mutant2.0 was trained to predict both the sign and the value of free energy change and it uses pH, temperature, neighboring residues and solvent accessibility in addition to mutation data. Although it achieves less accuracy compared to I-Mutant (0.62–0.80 depending on the input, whether sequence or structure-based are used, and the types of datasets), its ability to predict from protein sequences and not necessarily requiring structural information is the main advantage.

The MuPro server [23] also use SVMs to predict the sign of free energy change or the value of free energy change from sequence or structure-based input. A local window centered on the mutated residue is used as an input, which enables a direct use of the sequence information to the SVM. Superior correlation (0.86) with the experimental data was obtained compared to I-Mutant [31] as well as FoldX (0.75), DFIRE (0.68) and PoPMuSiC (0.85). Another server named Machine Learning for Protein Stability Changes (MLSTA) [39] utilizing SVM together with evolutionary features and different integration techniques, in which both sequence and/or structure-based input can be used, achieved slightly higher accuracy (0.84–0.90) than the machine learning methods described so far. In a more recent study [40], the sequence-based data with evolutionary properties were combined together with predicted structural features to make predictions upon mutations. This method achieved a slightly higher correlation coefficient than the MLTSA obtained. A subsequent study [41], utilized the same approach with a modified dataset and in result, obtained slightly better accuracy than all the methods previously described. It is suggested that the previous studies might be overestimating the correlation between the calculated and experimental stability because of the nature of the databases that were used. Therefore, it was argued that a fair evaluation can only be achieved by excluding different mutations of the same protein and only including proteins that have low sequence similarity in the training datasets. Aside from that, this method included evolutionary and predicted structural features as well as physical amino acid parameters. As a result, 66% and 74% accuracy was achieved in determining the stabilizing and destabilizing mutations, respectively. Also, a correlation of 0.51 was obtained in comparing the value of the change in free energy upon mutations. The results were found to be superior in comparison with modified versions of MuPro and I-Mutant2.0, *i.e.*, predictive properties added accordingly, using the same dataset.

In addition to the studies briefly reviewed above, there are a number of other studies and webservers available that utilize machine learning approaches to decipher the stability changes due to mutations. Namely, these servers are ESLPred [42], SVMProt [43], svmPRAT [44,45], Automute [46,47], FISH [48,49], onD-CRF [50], proSMS, PROTSRF, iPTREE-STAB [51], MuPro, SCide [52,53], SCpred [52], MuStab [21,22], PopMuSiC, PMut [54,55,56], SNAP [57], SNPs and Gene Ontology (SNPs&GO) [58], Parepro [59], CanPredict [60], nsSNPAnalyzer [11,61,62], MutPred [24], Hansa [63,64], Mutation Taster [19] and BeAtMuSiC [65].

Besides studying the protein stability changes upon mutations, the sequence-based machine learning methods are also used for analysis of the effects of mutations on the subcellular localization of the corresponding proteins. It is anticipated that proteins function properly if they are in their native localization, although some exceptions do exist [66,67,68]. In about 1% of cases, nsSNPs occur at a signaling region that may cause protein subcellular delocalization [66,67,68]. The change in protein subcellular localization due to mutations often disrupts normal cell function by changing protein concentrations [66,67,68]. Therefore, these mutations are closely associated with phenotypes. Several computational tools are available to predict the protein subcellular localization as reviewed below. Most of the methods that predict protein subcellular location are sequence-based and utilize machine learning. The idea is to represent the proteins in a classifiable manner based on sequence and use a set of proteins with known subcellular locations to train the computer, and then the method can be used to predict subcellular locations of new sequences after the approach is tested with a control dataset.

The protein subcellular location predictors mainly differ in several aspects including (1) the coverage scope, *i.e.*, how many subcellular locations are covered; (2) multiple or single-site cases, *i.e.*, whether proteins with multiple subcellular locations are predicted or not; (3) training dataset construction, *i.e.*, the amount of sequence identity is tolerated in the database; (4) organism-specific approach, *i.e.*, whether the approach is for multiple organisms or organism-specific; (5) representation of the protein sequence; and (6) prediction algorithms such as classifiers used in machine learning and testing algorithms [67]. In general, the desirable features are the wide coverage, inclusion of multiple sites, reduced sequence identity in datasets to exclude the homologous effects, rigorous protein representation method and organism-specific approach to increase the accuracy of the application along with solid learning and testing algorithms.

Since the earliest subcellular predictors PSORT [69], SignalP [70,71] and TargetP [72], there has been substantial progress in the development of these predictors. One of these, Hum-mPLoc 2.0 [73,74,75], which is a human subcellular localization predictor, shows a substantial advancement over earlier versions, the Hum-Ploc and Hum-mPLoc. Briefly, the datasets included only proteins with 25% or less pairwise sequence identity, 14 localization classes were covered, and both single and multiple-site proteins can be predicted. Their method hybridizes a higher level GO approach [76] and an advanced pseudo amino acid composition method [75,77]. The accuracy reached is 63%. This method has recently been improved [78] and the server Gene Ontology Annotation SVM (GOASVM), which achieved 72% prediction accuracy, was made available.

In another tool, LocTree2 [79], cellular protein sorting mechanisms are mimicked by utilizing SVM with a decision-tree like architecture of localization classes. A similar approach was also used in LocTree [80]. Eighteen localization classes are covered for eukaryotic proteins in LocTree2 as opposed to six in LocTree. Also, the sequence bias was reduced compared to LocTree by using the UniqueProt approach [81]. This corresponds to a threshold of 20% and 25% sequence identity for sequences longer than 250 amino acids for the development and testing datasets, respectively. The highest accuracy reached is 65% (eukaryotic proteins), showing comparable performance to the Subcellular Localization Predictor (CELLO v. 2.5) [82,83] and Wolf PSORT [84] for 52 eukaryotic proteins and 201 human proteins; the accuracy is 40% for the latter.

Recently, a Naïve Bayesian Classification (NBC) method was used utilized in n-gram-based Bayesian Localization Predictor (ngLOC) tool [85], which uses only the sequence information and is capable of predicting 11 sites for eukaryotic sequences with a high accuracy rate (89% for animal and 91% for plant proteins). Its performance was compared to SherLoc2 [86] (predicts 11 sites, requires sequence and text-based input together with phylogenetic profiles and Gene Ontology (GO) terms) and WegoLoc [87] (predicts 10 sites, uses sequence and weighted GO input). The ngLOC was found to outperform SherLoc while showing a comparable performance compared to WegoLoc. Since ngLOC only needs sequence information as input, it is applicable to a broad genomic data. However, it was noted that the performance of the method strongly depends on the size and the similarity in the datasets used [85]. Other predictors include pTARGET [88,89,90] and YLock [91,92]. A more comprehensive list of similar webservers and other methods without webservers are listed in the PSORT website (http://www.psort.org). Here, we focused on predictors for eukaryotic proteins and refer the reader to other reviews for other organisms and also for more information [66]. In addition, it is worth mentioning a recently released database named COMPARTMENTS [93], which is a unified, comprehensive database of protein subcellular location with all protein identifiers and GO terms.

Another area where the machine learning approaches are widely used is the prediction of folding rate changes caused by mutations. In one of these studies, the PRORATE webserver [94], a structure-based method using machine leaning is implemented to predict the folding rate changes upon mutations. In this approach, structural topology parameters with the complex network properties are used as the input features for support vector regression, which are then used to calculated the folding rates [94]. The method was shown to reach a correlation coefficient of 0.90 with respect to experimental folding rates. Other servers for calculating protein folding rates include K-Fold [95], FOLD-RATE [96,97,98], Prediction of long-range Contacts (PROFcon) [99] and Protein Property Prediction and Testing Database (PPT-DB) [100].

Similarly, machine learning methods can also be used to predict protein aggregation properties. For example, the Protein Aggregation Prediction Server—Random Forest (ProA-RF) and ProA-SVM servers [101] utilize a machine learning-based method. Out of 560 physico-chemical properties, 16 were identified as important to protein aggregation, which then were used to make new predictions. These 16 features include hydrophilicity of polar amino acid side chains, various descriptions of hydrophobicity, accessibility reduction ratio, shape and surface features of globular proteins among others. The ProA was shown to outperform several other sequence-based predictors, namely, Waltz [102], PAGE [59], FoldAmyloid [103] and ZYGGREGATOR [104].

Considering the growth of data size in genomics, machine learning stands out as a substantially efficient approach. Besides efficiency, machine learning methods have an advantage over biophysics-based approaches due to their ability to learn complex nonlinear functions from mutation information to sequence and structure. However, they cannot predict the molecular mechanism of the effect and will fail in predicting mutations that are not observed in the training database. Because of that, biophysics-based approaches are very much needed.

### 2.2. Statistical Potentials

The statistical potential energy functions are an alternative to the energy functions delivered from first principles, *i.e.*, biophysics-based energy functions. Any structural characteristic in the 3D network of interactions in folded structures can be incorporated in the derivation, where these characteristics are converted to the corresponding free energies (potential of mean force) through Boltzmann statistics [105,106,107]. Despite their simplicity, these knowledge-based potentials have been used with considerable success in mutation-induced stability change predictions and many other applications [65,108,109,110,111]. Over time, the features that incorporate multi-body effects and collectivity have been added to increase the accuracy of predictions [108,112,113,114,115]. Although this can be considered an advantage over pairwise first principle-based potentials, statistical potentials are inheritably approximate because the statistics are collected from unrelated proteins, which implies that different structures used all belong to the same thermodynamics ensemble [116]. These approximations may result in a database-dependence in the delivered potentials [117]. Different statistical potentials such as distance-dependent potentials, distance-independent contact energies, backbone torsional potential and solvent accessible potential differ in how the statistics are calculated. Also, depending on the level of details included, there are coarse-grained residue level and atomic level potentials [116].

Among the most popular statistical potentials is the DFIRE [35], which is a distance-dependent, pairwise statistical potential. The DFIRE has been developed in search of a transferable, pairwise potential that is built on a physical basis with few or no adjustable parameters. Its database dependence, ability to capture 20 amino acid characteristics, solvent exposure dependence, transferability and accuracy in different applications were tested. It produced superior results in comparison with two other statistical potentials RAPDF [118] and KBP [109]. In addition, it was found to achieve a success rate of 70% compared to RosettaDesign [119] and FoldX [33,34]. Its poor performance in a few cases [35] can be attributed to the fact that it is a pairwise potential that only depends on distance, and also the solvent effects, polar–polar interactions and hydrogen-bonding are taken onto account only implicitly. Subsequent improvements include the development of DFIRE2 [36] and dDFIRE [35], which are based on orientation dependent interactions by treating each polar atom as a dipole with a direction. The dDFIRE and DFIRE2 servers produce a protein conformational free energy score based on provided structures.

In parallel with the DFIRE development, another statistical potential, the Site Directed Mutator (SDM) [110] server, was developed to predict the stability changes due to nsSNPs. In the first test case, using the same set of input proteins as PoPMuSiC2, the SDM performed comparably, but not better than a number of other methods used in comparison when predicting whether a given mutation is stabilizing or destabilizing [110]. Other servers based on statistical potentials include Cologne University Protein Stability Analysis Tool (CUPSAT) [111] PopMusic2.0 [38], AUTOMUTE [46], BeAtMuSiC [65], MuPro[23], MuX-S [120], MuX-48 [23] and Hunter [1,121,122].

Aside from the statistical potentials, graph methods can also be used for screening large datasets. A recent study [123] used a graph-based method (mCSM server) in which distance patterns between atoms were used to represent the structural information of residues and to train the predictive models. mCSM was shown to perform significantly better than PoPMuSiC (1.0 and 2.0) [38], Automute [46], CUPSAT [111], Dmutant [113], ERIS [124], I-Mutant2.0 [32] and SDM [110]. Another graph-based server is the Bonds on Graphs (BONGO) server [125].

### 2.3. Biophysics-Based Methods

The statistical/knowledge-based potentials that were reviewed in the previous section can be replaced by physics-based empirical potentials (energy components). In this approach, typically, a linear interaction energy based formulation [126] has been used to calculate the binding free energy or folding free energy differences between the mutants and the wild type. A typical form of the free energy change as commonly used recently is as follows [127,128,129,130]:

*G* = α (∆*V*_vdW_) + β *(∆V_elec_)* + γ *(∆SA)* + δ (1)

In this formulation, the contributions of van der Waals energies, electrostatic energy (Coulombic and polar solvation energy) and nonpolar component of solvation energy (a term based on solvent accessible area) are calculated based on wild-type and mutant structures. Note that the general formula should include internal energy, an estimation of entropy and other energy terms, but various investigations have omitted them based on some assumptions or how they affect the overall performance of the method [127,128,129,130].

**Figure 2:**
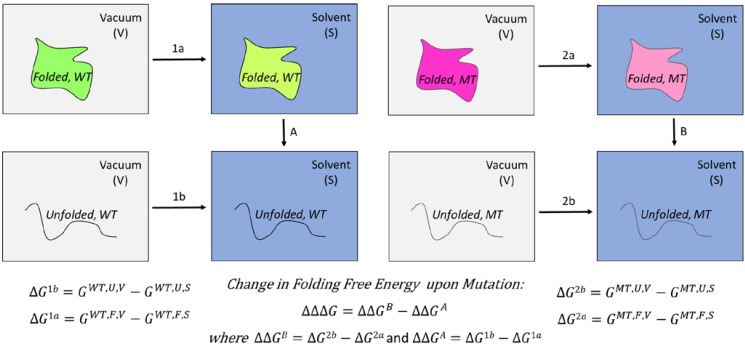
Schematic diagram of different states involved in the energy calculations and the corresponding equations used to predict the change of the folding free energy upon mutation. WT, Wild-type; MT, Mutant; Green: Folded WT protein in vacuum (V, gray); Light Green: Folded WT protein in solvent (S, blue); Violet: Folded MT protein in vacuum; Light Violet: Folded MT protein in solvent, Unfolded (U) states are represented by black curved lines.

The parameters in the linear interaction energy (LIE) equation are optimized by fitting the predicted energy changes against large databases of experimentally determined charges of folding or binding free energies upon mutations. The free energy is calculated using the thermodynamic cycle shown below. Therefore, the overall free energy change is calculated in a path-independent manner (Figure 2).

Previous studies that used this approach differ in three aspects; how the initial structures for the wild-type and mutants are generated, which contributions to the free energy are taken into account and how the unfolded state is modeled. In one of these studies [128], an ensemble of initial configurations generated by the program Concoord [131] were used as initial structures. Also, the polar and nonpolar contributions to solvation energy were calculated through an efficient continuum solvent approach by solving the Poisson-Boltzmann equation using the DelPhi package [132] which then was averaged over the structural ensembles. Also, the entropy was calculated based on a quasiharmonic approximation proposed by Schlitter [133]. The final free energy equation included the electrostatic, van der Waals, solvent accessible surface area, and entropy terms with three adjustable parameters and a constant. This method named Concoord/Poisson-Boltzmann surface area (CC/PBSA server) produced a correlation coefficient of 0.75 for seven proteins and 582 mutants, which was compared to results from FoldX (*R* = 0.73, with five adjustable parameters) and Eris (*R* = 0.75, with 20 adjustable parameters) [128]. More recently, a similar approach [129] also used three adjustable parameters a constant, and in result, obtained a correlation coefficient of 0.72 for 10 proteins and 822 mutations. Energy minimized structures for wild-type proteins were used and the mutant structures obtained based on these wild-type structures only differed at the mutation site. The mutant residue side chain structure was modeled through a rotamer search. The unfolded state was represented using a 5-residue segment (mutant residue and two neighboring residues on each side) from the folded structures.

Another recent study [134] used the method named scaled molecular mechanics generalized Born method (sMMGB) to calculate folding free energy differences for 1109 mutants (662 of them were used to fit the weights of each contribution to the energy). The free energies were calculated through the GB method in TINKER software [135] using three different molecular mechanics force fields and then averaged. The resulting free energies were scaled with a linear coefficient and a constant using the experimental free energy values of 662 mutants. The results were found to be comparable to those from Eris, FoldX and I-Mutant. In addition, the unfolded state was modeled with 3, 5, 7, 9, 11 and 13 residue segments with the mutant residue in the middle and not surprisingly the smallest one provided best results. This is because of the fact that this approach assumes the same unfolded state for the rest of the protein except the segments considered and this assumption is expected to hold better with smaller length segments.

Most recently, a similar physics-based linear interaction energy approach named modified MM-PBSA (molecular mechanics Poisson–Boltzmann Solvent Area) [130] was used to predict the protein stability changes upon mutations using large datasets (several thousand mutations). The binding free energy was composed of van der Waals energy, polar component of solvation energy and the solvent accessible surface area in the binding interface. Effects of using different minimization schemes and using different dielectric constants for solvation energy component were also assessed. The predicted energies produced the highest correlation constant of 0.69. This method provides fast predictions that are applicable to large datasets.

Other resources that also use similar physics-based energy approach to calculate the stability changes upon mutations are the methods and webservers; Eris [124], FOLDEF [136], EGAD [137] and FoldX [33,34].

In principle, the statistical mechanics based methods such as molecular dynamics (MD) give the most detailed information about the biological systems studied. The all-atom MD simulations produce the physical movements of the atoms in a system by numerically solving Newton’s equations of motion. As a result, a number of different properties can be obtained from the ensemble of structures generated by MD such as H-bond network, flexibility of the protein (based on fluctuations), and radius of gyration. In a recent MD study [138], the observed conformational changes in the mutant F28L compared to the wild-type structure of RAC1 protein in a ~300 ns long MD simulation. It was shown through the RMSD, RMSF and H-bond network change that the mutation caused loss of native conformation in the Switch I region, which is associated with its oncogenic transformation.

Although detailed structural and dynamical information about the proteins can be obtained through the MD simulations, the direct calculation of free energy from the standard MD is possible through more advanced methods. In principle, the most accurate methods to calculate free energy differences include free energy perturbation (FEP) and thermodynamic integration (TI), which use the trajectories from MD or Monte Carlo (MC) simulations [139,140,141,142]. First, a real or alchemical pathway is defined from one state to another. Then, in the TI approach, the free energy change between the two states is calculated by integrating over enthalpy changes along the path. On the other hand, in the framework of the FEP approach, the free energy difference from state A to B is obtained using Boltzmann sampling through the equation; ∆*G* = −*k*_B_*T*ln (*e*^−(*E*_B_−*E*_A_)/*k*_B_*T*^), where *k*_B_ is the Boltzmann constant and *T* is temperature.

Although, the coupling of these methods with several advanced sampling approaches increases the efficiency of these methods, they still are not suitable to study large datasets of mutants. Therefore, more efficient methods, as summarized in this section, based on approximations to the free energy have been developed to predict the protein stability changes that are applicable to large databases of mutants.

In addition for studying the free energy changes, the biophysics-based methods were used to study the protein kinetics such as protein association/dissociation rates, protein folding rates and aggregation rates. Apart from being exhaustive, we will briefly review representative studies in each of these aspects, starting with the TransComp server [143], which is based on the transient-complex theory [144] for predictions of protein–protein and protein-RNA association rate constants. In this approach, a two-step reaction mechanism is assumed as shown below (Equation (2)), in which the transient-complex, A*B, is first formed and then the functional dimer. The two proteins have a near-native separation and right orientation at the transient-complex but yet to form specific short-range interactions of the native complex.

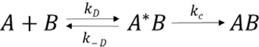
(2)
where, the overall rate constant is *k*_a_ = *k*_D_*k*_c_/(*k*_−D_ + *k*_c_). At this point, the method assumes that the association is diffusion limited as opposed to conformational rearrangement (*k*_D_ >> *k*_c_ and also *k*_c_ >> *k*_−D_), therefore once the transient-complex is formed, the reaction proceeds to form the native complex. The rate constant for the formation of the transient-complex by random diffusion is called the basal rate constant, *k*_a0_ and the overall association rate constant is calculated by *k*_a_ = *k*_a0_e^−∆G_el_*/*k*_B_*T*^ where ∆G_el_* is the electrostatic interaction free energy of the transient-complex. The diffusion is efficiently modeled through Brownian dynamics, and the electrostatic free energy is calculated through the Poisson–Boltzmann equation. Again, this method works for diffusion limited cases that correspond to cases where the association rate constant ≥~10^4^ M^−1^·s^−1^ (the full range of association rate constants span a range of 1–10^10^ M^−1^·s^−1^). Single point mutations are not expected to make a significant change in terms of the random diffusion because the proteins are treated as rigid bodies with no charge for efficiency during this process, therefore, the accuracy of the change in rate constants of mutants with respect to wild-type structures strongly depends on the electrostatic free energy calculation.

Other approaches have also emerged from the need for a fast and widely applicable method. In general, methods like standard MD or biased MD are either limited by time and/or the complexity of the procedures. A structure-based method was developed [145] to predict both dissociation and association constants along with equilibrium rate constants. The structural features were determined based on a diverse dataset of 62 protein complexes with known experimental rate constants. This method uses structural information as only input and linear models were developed for predictions based on the structural input using Bayesian information criteria [146]. In result, the method produced correlation constants of 0.80, 0.73 and 0.77 for *k*_off_, *k*_on_ and *k*_D_, respectively.

Recently, a method [147] based on hotspots data was developed to predict the change in protein–protein dissociation rates upon interface mutations. In general, hotspots are defined as a set of interface residues that destabilize the protein changing the binding free energy by 2 kcal/mol or more upon being mutated to alanine [148]. In this approach, a set of descriptors are generated through machine learning using the data from alanine scans of hotspots and hot regions in relation to changes in dissociation rates upon mutation. As a result, a mutation to any residue type can be studied as well as multi-point mutations with an accuracy of 0.79 compared to experimental off-rates.

Single point mutations also affect protein/peptide aggregation kinetics depending on their effects on the protein biophysical characteristics. In a simple approach [149], the changes in hydrophobicity (∆*I*^hydr^), secondary structure propensity (∆*I*^SS^), and charge (∆*I*^ch^) were related to the ratio of wild-type and mutant protein/peptide aggregation rates (log (*v*_wt_/*v*_mt_)) with coefficients obtained by fitting to experimental values as shown below (Equation (3)) with three adjustable parameters α_hydr_, α_SS_, α_ch_:

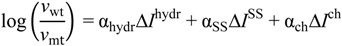
(3)


Despite its simplicity, this type of consideration produced a correlation coefficient of 0.8 with experimental and predicted aggregation rates for a series of proteins and peptides.

Another simple approach [150] that utilized a simple mathematical expression with no adjustable parameters produced a correlation coefficient of 0.85. The method is based on amino acid properties at the mutation site, total charge and β-propensities as shown below (Equation (4)):

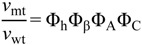
(4)


where, Φ_h_ is the ratio of solvent accessible surface area of mutant residue to wild-type residue or *vice versa* depending on whether the mutation is from apolar to apolar, *i.e.*, no dipole or charge in side chain or polar to polar. If the mutation is from polar to apolar or *vice versa*, then dipoles are used. The second factor (Φ_β_) is the β-propensity of mutant to wild-type. The last term, Φ_A_Φ_C_, approximates the effect of aromatic residues (A) and total charge (C); Φ_A_Φ_C_ = *e*^(Δ*A*−Δ|*C*|/2)^.

Another method, the TANGO server [151,152,153], used a statistical mechanics algorithm based on the physico-chemical characteristics of secondary structure formation with the assumption that the core regions of aggregates are fully buried. The TANGO algorithm basically calculates the partition function of the phase space, where every segment of each peptide is allowed to populate different conformational states according to Boltzmann distribution to form aggregates. The energetic penalty for complete desolvation of the core regions in aggregates involves contributions from solvation, van der Waals, H-bonding, entropy and electrostatics. As a result, solvation propensities of 179 peptides were correctly predicted with a correlation coefficient of 0.74.

Moreover, a method, named Prediction of Amyloid Structure Aggregation (PASTA) [154,155] was developed to calculate sequence-specific interaction energies between pairs of protein fragments using statistical analyses of the native folds of globular proteins. In parallel to PASTA, the AGGRESCAN [156] webserver was developed, which calculates the aggregation propensities based on the predetermined aggregation propensities through experimental data of each amino acid in given sequences.

## 3. *In Vitro* Analysis of Pathogenic Mutations

SNPs encompass a high density distribution of genetic variation in the human genome. Each SNP represents a difference in a single nucleotide, including transition, transversion, insertion or deletion between individuals of a biological species or alleles in the paired chromosomes. Since 1994 SNPs have been proposed as the third generation of genetic markers, as well known as the restriction fragment length polymorphism (RFLP) and simple sequence repeats (SSRs) [157,158]. Over 10 million SNPs have been found at a frequency of 1–10 in 1000 base pairs through the whole human genome, which may affect individual development and reflect human evolution. Immediately following their discovery, SNPs attracted the attention of more and more researchers. SNPs contributed to the susceptibility for complex diseases based on the statistical genetics [159], and make whole-genome association studies more feasible, e.g., Alzheimer’s Disease, Breast Cancer and Coronary Heart Disease [160]. In this section, we will review the most common and newly developed detection methods and functional screening for SNPs.

### 3.1. Single Nucleotide Polymorphisms (SNPs)—Detection Methods and Technology

During the past decade, new detection methods and technologies with high sensitivity were introduced to help detect SNPs more efficiently, such as Next Generation Sequencing (NGS), restriction fragment length polymorphism, and random amplified polymorphic DNA. At the same time, a prototype of the SNP database was established that compiled the contributions from many different research groups. However, most of the traditional technologies were based on the detection of gel electrophoresis, which are difficult to perform in high-throughput and multiplex format. The development of SNP microarray technology makes SNP detection highly efficient, fully automated, and relatively inexpensive. It is even possible to apply SNPs as aids for molecular diagnostic, clinical examination, drug design and individual medical diagnosis. To collect and catalog the molecular variation within SNPs, researchers also submit their data to a SNP database, an online resource that is designed to contain all identified genetic variation. By accessing the database, researchers are not only promised to advance the variety of genetically based natural phenomenon, but also investigate evolutionary relationships. However, no single method meets the needs of all studies. In the next section we will discuss major methods and technologies that are widely used.

#### 3.1.1. Traditional Methods for SNP Genotyping

To scan for new polymorphisms and identify known gene variants in target genome, researchers have developed a number of technologies driven by the Human Genome Project. By referencing against the completed human genome sequence, DNA sequencing to the target genome is the most direct and accurate method. It is used as the gold standard for SNP genotyping. Like others, the DNA sequencing technologies are evolved from a single but complicated method to a variety of automated methods to meet different requirements. The development of next-generation sequencing technologies promoted whole genome SNPs screening at levels not previously possible [161,162]. As it has become more widely accessible, usage has extended from basic research into clinical contexts. The challenge is not genome sample preparation or the cost, but how to analyze the newly generated data and how to handle the rising error rate. The NGS technologies lead sequencing base call accuracy range from 99.9% to 99.9999% [163]. Even for the lowest error rate, the absolute number of miscalled genome variants is still unwieldy. Indeed, post-processing techniques to help reduce the uncertainty in the final genotyping variant call including *K*-Spectrum approach, Suffix Tree/Array approach, Multiple Sequence Alignment approach and Hybrid approach have been developed [163]. Careful sample selection and preparation may save more time and cost for a given gene based on these two broad areas; known SNP identification and unknown SNP exploration. For both global and regional target genomes, DNA sequencing followed by various gene amplification approaches are suitable for detecting known and unknown SNPs, but more options are available when screening known SNPs in individual genomes, especially for disease patients. Two classical methods are discussed below.

**Figure 3:**
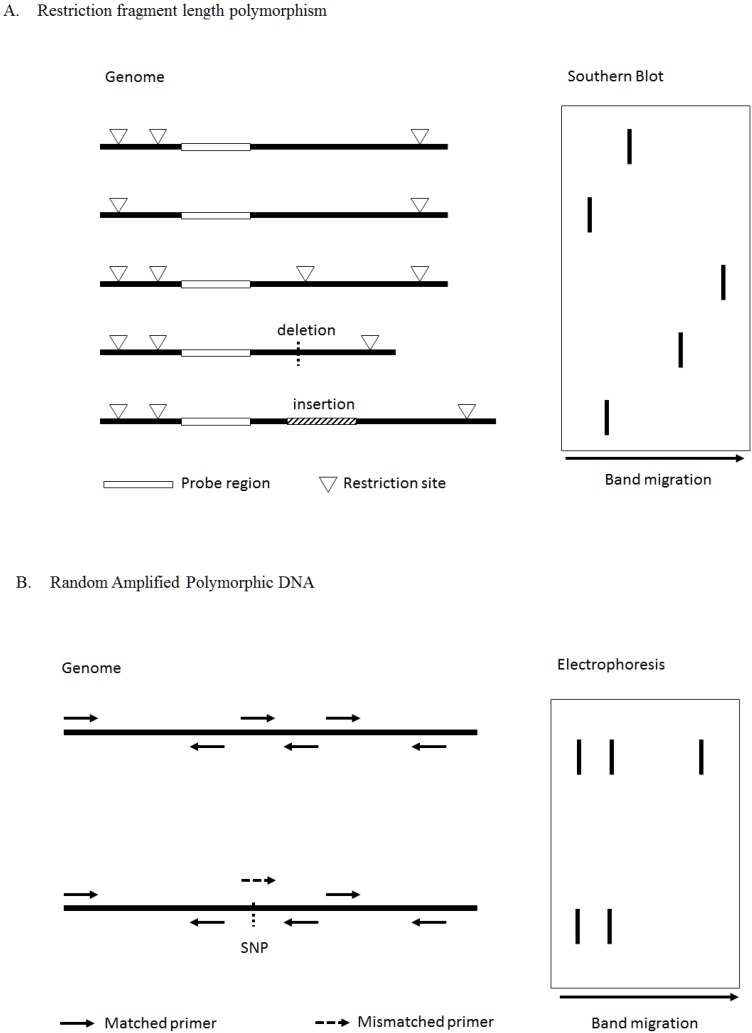
Principles of Restriction Fragment Length Polymorphisms (**A**) and Random amplified polymorphic DNA (**B**). The resulting fragments from target genome DNA are generated (**left**) and separated by gel electrophoresis followed by appropriate detection approach (**right**). To perform restriction fragment length polymorphisms (RFLP), genome DNA from variant individuals are subject to restriction digest (restriction sites are indicated by triangle) and random genomic probe (complement sequences are indicated by empty rectangle) is used for Southern Blot detection. Bands shown in Southern Blot represent (1) wild type; (2) loss of restriction site; (3) gain restriction site; (4) deletion of DNA fragment; (5) insertion of DNA fragment, from top to bottom. To perform RAPD, random primer sets are used to amplify genome DNA. Each matched primer set (full line arrow) generates a specific size product that appears in both wild type (**top**) and mutant (**bottom**) genome. Meanwhile mismatched primer (dashed line arrow) causes band absent in mutant.

The first SNPs found in a global approach were restriction fragment length polymorphisms (RFLP) [164]. As described in Figure 3A, high quality genomic DNA from multiple individuals are digested with selected restriction enzymes, then the resulting fragments are separated by gel electrophoresis and transferred to nylon filters. A random genomic probe is used for identification of variation in the restriction fragment lengths. Only SNPs in the restriction site or in the probe sequence can be detected. The introduction of Restriction Fragment Length Polymorphism Analysis of PCR-Amplified Fragments, in which the primer was designed with an additional mismatch base adjacent to the SNP site did not, unfortunately, improve detection [165]. However, RFLP is a simple and low-cost method to detect known SNPs. For example, a T to A transversion in the middle position of codon 6 of hemoglobin causes sickle-cell disease. This mutation generates the sequence 5'-CTGAGG-3' that is recognized and is cut by *Dde*I [166], and a short fragment can be detected in the patient genome.

Random amplified polymorphic DNA (RAPD) is the first PCR based molecular marker technique developed [167] and then applied to SNP detection for various organisms [168,169]. Random and variable sequences of genome PCR products are generated by random primer sets and produce a pattern of bands resulting from electrophoresis. Presence/absence of certain size product fragments indicates individual variety (Figure 3B). Mismatches between the primer and the template resulting in the absence, or decreased transcripts of specific bands, indicates the position of a SNP. By designing longer specific primers, the product DNA can be sequenced to identify the gene variants.

Needless to say, these classical methods are laborious and time consuming. High-throughput and multiplex analysis is required for whole genome SNP mapping and individual SNP detection. SNP microarray technology using a high-density oligonucleotide probe array makes it possible to perform whole genome SNPs genotyping. The microarray platform enables microsphere-based microarray [170], fiber-optic microarray [171] and glass substrate microarray [172]. The marker density among the various technologies ranges from hundreds to millions. In the Lettuce Affymetrix SNP Array, for example, 6.4 million markers, spread evenly across whole genome can be simultaneously analyzed [173]. Thus, the bottleneck for high-throughput SNPs detection is no longer the microarray platform, but the methods and technology of DNA preparation.

#### 3.1.2. High-Throughput Method for SNP Genotyping

Allele-Specific Primer Extension (ASPE) is a solution based, sequence specific enzymatic reaction technology allowing the analysis of multiple SNPs in a one-step single reaction [174]. To determine the target genotype, a pair of allele-specific primers for each mutant or polymorphic site that differ at their 3' end, complementing to either of the variant alleles, are immobilized at their 5' end onto the array. Extension of the detection primers with labeled dNTPs is then performed in a template-dependent manner. To evaluate the result, an appropriate array scan is performed to quantify and compare between each primer pair by labeled probe. The signal ratios fall into distinct categories defining the genotype at each site. However, the caveat for this method is that microarray is not reusable and the extension reaction is technically challenging to perform on the microarray. For these reasons, a variety of changes to the original method have been made, one of which, a modified two-phase procedure, is described here [175]. Instead of 5' end immobilized on microarray, a capture sequence is used to recognize and capture the target onto the solid microsphere-based microarray. The capture phase is followed by the genotype determination phase. Taking advantage of this modification allows sequence labeled microsphere arrays to be used for new template detection. Most recently, the major histocompatibility complex (MHC) class I chain-related gene A (*MICA*) genotyping was developed by ASPE on microarrays [176]. By using 20 control primers, strict and reliable cut-off values were applied to select high-quality specific extension primers. Fifty-five allele-specific primers were selected as optimal primers, in which forty-four primers could be initially used. On the basis of showing the same results as those by nucleotide sequencing, ASPE on microarrays provide high-throughput and also high accuracy genotyping for *MICA* alleles used in population studies, disease-gene associations and hematopoietic stem cell transplantation, although overmuch primer designation is still required for gene specific optimization.

Based on the principle of ASPE, a commercialized product called GoldenGate™ [149] allows up to 10^6^ multiplex for custom SNP microarray by using only 250 ng genomic DNA. For each SNP locus, a pair of allele-specific oligonucleotides and one locus-specific oligonucleotide is designed. All three oligonucleotides contain sequence complementary to genomic DNA and also universal PCR primer sets. The locus-specific oligonucleotide is designed complementary to the downstream sequence 10–20 bp from the SNP site, to prevent repeat sequence as well as palindromic sequences and increase the specificity of the reaction. DNA polymerase is used to fill the gap between the two kinds of oligonucleotides. The resulting product is used as a template for amplification by universal PCR primers with various fluorescent labels followed by hybridization and detection as described for ASPE. GoldenGate**^TM^** provides high throughput genotyping at the multiplex levels that fulfills the requirement of custom genotyping, and also the accuracy can achieve 96.64% [177,178]. In a study [179], GoldenGate™ offers substantial increases in accuracy for pooling compared to other commercial microarray technology, and this advantage greatly extends the usefulness of pooling, making the limitation of whole genome studies the available sample size, rather than cost.

Single Base Extension (SBE) technology is a robust technology that allows for identification of a known SNP site and is commonly used for low-density array development and DNA preparation. It was invented by Philip Goelet, Michael R. Knapp and Stephen Anderson in 1999 to measure DNA methylation levels in the human genome [180]. The method is to design a dual functional DNA oligomeric primer in which the 3' end complements to the locus that is to be genotyped and the 5' end complements to a sequence tag. In the presence of DNA polymerase and labeled ddNTPs, the 3' end of the primer appends a single DNA base and undergoes amplification exponentially. The identity of this appended base indicates the genotype of the original template. Since each locus has a distinct tag, the genotyping reactions can be performed as highly multiplexed and separated by hybridization to the reverse complement sequence tags on the microarray. This method allows researchers to indicate SNPs by using universal DNA arrays such as all *k*-mer arrays, and provides a flexible, high-throughput and cost-effective alternative to genotyping assays [181]. Previously, over 100 SNPs were genotyped and over 5000 genotypes were obtained by using SBE microarray technology with only an approximate 1% error rate [182]. Based on a similar principle, SNPstream ultra-high throughput technology was developed by using multiplexed PCR (up to 12-plex PCR sets) in conjunction with SBE genotyping technology. Single nucleotide appending is manufactured in a 384-well format on a glass-bottomed plate [183]. Because two different fluoresceins are labeled for each one PCR primer set, only one genotype can be detected in a single reaction, which limits the application of SNP stream in clinical practice, and this technology is not readily multiplexed for high-throughput applications.

TagMan probe arrays are designed based on fluorescence resonance energy transfer (FRET) to quantify nucleic acids. It combines the advantages of real-time quantitative PCR and gene expression microarray methods, and reduces their limitations. A conventional TagMan probe is designed as a double-labeled fluorogenic probe, the donor fluorescent dye is attached to the 5' end and the acceptor fluorescent dye is attached to the 3' end. With 5' nuclease activity of DNA polymerase, the donor will be cleaved during the extension step of the PCR, which reflects single nucleotide un-pairing by evaluation of FRET efficiency. The modified TagMan probe consists of an amino group at the 3' end for immobilization, poly(T)_20_ as a linker arm with a reporter (donor) fluorescent dye, and a dabcyl group at 5' end as the quencher (acceptor). The modified TagMan probe is immobilized onto a glass-based microarray so that a real-time reporter signal restoration can be detected by cleavage of 5' end quencher during the PCR to represent the feasibility of real-time nucleic acid analysis in parallelism directly from genomic DNA [184]. Although TagMan probe arrays have advantages in specificity, sensitivity and dynamic changes of fluorescence intensity at various PCR cycle numbers for SNP analysis, the specificity is significantly impacted as multiplexing increases, because PCR amplification and TagMan probe cleavage are performed in a single reaction [185].

The Ligation-rolling Circle Amplification (L-RAC) is a flexible, non-gel-based SNP detection method. Circularization of padlock probes with T4 ligase or thermostable ligase is used to discriminate point mutations in target DNA sequences specifically and sensitively. A short padlock probe includes the sequences at both 3' and 5' ends, which are complementary to downstream and upstream sequences of target SNP in a single copy gene. The probe will be circularized by a high fidelity thermostable ligase, or else fail in ligation causing by a single base mismatch at the 3' end. The other element of the short padlock probe contains a tag sequence and restriction enzyme site. By rolling circle amplification and enzyme digestion, the resulting products can be used for hybridization analysis [186]. A scalable method [187] based on L-RCA was reported to analyze numerous target sequences in multiplexed assays. All 13 sets of padlock probes tested were co-amplified and identified by hybridization to standard tag oligonucleotide array for analysis SNP among patients with Wilson’s disease [187]. The key feature of L-RCA, unimolecular dual recognition probes, allows lower probe concentrations that reduce the risks of amplification artifacts. On the other hand, the design requirements of the probes also limit the applications in high-throughput genotyping analysis.

Molecular Inversion Probe (MIP) is derived from L-RCA, in which the linear oligonucleotides were used as padlock probe sets providing sufficient specificity. An additional exonuclease treatment removes non-reacted linear probes, and thus avoids cross-talk at the detection step. The MIP is designed with seven functional components: two restriction endonuclease enzyme recognition sites (e.g., *Hin*dIII, *Bam*HI, *Eco*RI, *Eco*RV and *Xba*I); two regions complementary to the target genomic DNA, downstream and upstream to the SNP site respectively, at each termini of the probe; two general PCR primers common to all probes and one universal tag sequence for each locus. To start the procedure, linear probe is circularized after matching to target genomic DNA via an appending base at the 3' end, which matches the SNP site. Successfully reacted probes are linearized, released from the genomic DNA and amplified using the primer set. The resulting product containing inversed sequence is captured with the tag sequence. The multiplexing level ranges from 10^2^ to 10^6^, based on the capture platform [188]. However, four parallel reactions are usually required for each SNP variant, and longer probes are required for large gaps, which increases the cost required to achieve the expected sensitivity and uniformity.

Affymetrix SNP array technique became popular and is widely used for whole-genome scans of polymorphic genetic markers. An algorithm was designed and developed by Affymetrix for genotyping SNPs based on the intensities, and the derived genotypes are available for further analysis. Prior to the bioinformatics analysis, the whole genome is fractionated with selected restriction enzymes and the resulting fragment lengths range from 200 to 2000 bp, followed by ligation of adapters to fragments, and a subset of the genome is amplified by single primer amplification reaction and is ready for hybridization. Various arrangements and combinations of the restriction endonuclease enzymes allows for multiplexing ranging from 10^4^ to 10^6^. A modification on this method, based on using multiple arrays at the same time and a model that relates intensities of individual SNPs to each other, allows for annotating SNPs that have poor performance, thus extending the application of the Affymetrix SNP array [189]. Obviously, by the limiting restriction enzymes and sites, this method is incapable of custom application, as SNPs cannot be specified. On the other hand, the high-throughput whole-genome coverage compensates for the limitation.

In summary, microarray technological advances help identify whole genome SNP data from multiple sources efficiently, especially for prokaryotes and other genomic sequences of low complexity. However, the efficiency and design of the amplification reaction are still limiting the appliance of microarray, when faced with genomes as complex as human genomes. The SNP database offers a solution for the overwhelming multiplex of human genomic DNA. By accessing a current SNP database, like dbSNP (http://www.ncbi.nlm.nih.gov/SNP/) and JSNP (http://snp.ims.u-tokyo.ac.jp/), researchers are able to perform physical mapping, population genetics, investigations into evolutionary relationships, not to mention being able to quickly and easily search for variation in the gene of interest. These data now allow researchers to investigate the functional SNP involved in human diseases.

### 3.2. Functional SNP Screening

As the studies based on SNPs advanced, researchers began to notice the increasing probability of false-positive results generated by traditional statistical methods. To solve this problem, a fusion of traditional and newly developed *in vitro* methods and techniques were introduced to identify functional SNPs from overwhelming candidate loci. Here, we will go over the most important methods and technological development as listed in Table 1. Then, we follow the historic timeline of an X-linked gene and explain methods that contribute to each research milestone (Table 2) and also provide an outlook for future directions.

**Table 1:** Important methods and technological developments for understanding various single nucleotide polymorphism (SNP) loci.

SNP Loci	Subject of Investigation	Methods
Non-coding region	Gene transcription	Chromatin immunoprecipitation; Electrophoretic Mobility Shift Assay; Dual-luciferase assay; Real Time PCR
	mRNA degradation	
	Gene translation	
	Gene expression	Immunoblotting; Enzyme-Linked Immunosorbent Assay; Immunofluorescence
Coding region	Protein structure	Crystallization; Nuclear Magnetic Resonance
	Conformation Stability	Circular Dichroism Spectroscopy; Intrinsic Fluorescence
	Kinetics	Binding assays; Enzyme assays

Most commonly, SNPs are found in the non-coding regions, or as synonymous SNPs in coding regions that do not alter the amino acid sequence of the expressed protein. In both cases, SNPs are invisible to the resulting phenotype and play a role only as a genetic marker. SNPs can have effects based on their distribution within the elements of the gene, which includes regulatory regions, five prime untranslated regions (5' UTRs), introns and exons and three prime untranslated regions (3' UTRs). To identify functional SNPs, various methods are applied to specific coding regions. In general, the regulatory region is made up of a promoter, an enhancer and other structural domains. The main functions of these regions are to control the transcription rate by binding of transcription factors, or bringing in a loop-like structure to enhance the transcription factor binding efficiency or repress the transcription as needed. SNPs located in this region may change binding affinity between the transcription factors and the DNA of the gene. Either strengthening or weakening binding affinity, or upregulating or downregulating mRNA transcription can regulate the level of protein expression. The 5' UTR, also known as the leader sequence, is directly upstream of the start codon in the mRNA and plays an important regulatory role in the translation of a transcript in different ways [190]. The SNPs in this region will in many cases impair the translation process. It is reported [191,192,193] that microRNAs (miRNA) interacting with the 3' UTR of mRNAs also play a role in regulating gene expression. For example, a SNP localized in the pri-miRNA or 3' UTR can regulate degradation of mRNA, so it can regulate the expression of the gene [193]. Although differing in mechanisms, SNPs localized in regulatory region, 5' UTR and 3' UTR, have same effect on the level of the gene expression. Therefore similar research methods are used to understand the SNP in that region for a specific gene.

**Table 2:** Methods used in MECP2 studies along with the historic timeline of research milestones.

Year	Results	Methods	Reference
1992	*MECP2* was firstly identified in human X chromosome. MeCP2 belongs to MBD protein family, and binds to methylated DNA	Immunofluorescence; Southwestern Assay	[194]
1997	MeCP2 acts as a global transcriptional repressor	*In vitro* Transcription Assay; Western Blot	[195]
1999	Four mutations of *MECP2* gene causing Rett syndrome were identified	Conformation-sensitive gel electrophoresis; DNA sequencing	[196]
2000	Missense mutations in MBD domain lost 5mC binding specific. Interruption of TRD domain lost repression to various genes	Southwestern assay; *In vitro* Transcription Assay	[197]
2003	*MeCP2* regulate *BDNF* gene by binding to promoter IV	EMSA; ChIP; Real-time reverse transcription PCR	[198]
2003	*MeCP2* compact chromatin via its three AT-hook domain	EMSA; Sedimentation Velocity; Electron Microscopy	[199]
2005	*MeCP2* bind to four-way junction DNA in a structure-specific methyl-CpG-independent manner	Gel-retardation assays	[200]
2008	Mutational hotspots have effects on MeCP2 stability	Fluorescence Spectroscopy; Circular Dichroism	[201]
2012	*MeCP2* bind specifically to 5-hydroxymethylytosine, enriched in the target gene promoter	Substrate Affinity pull-Down; Methyl-DNA-IP Sequencing	[202]
2013	Mutational hotspot R306C, proximal to the T308 Phosphorylation site, abolishes the binding to the NCoR complex	Phosphotryptic-mapping; Peptide binding assay; Immunofluorescence	[203]
2013	*MeCP2* compact chromatin via its three AT-hook domain	Real-time reverse transcription PCR; Immunofluorescence	[204]

Binding affinity between regulatory factors and gene elements is usually investigated to understand regulation of the gene expression. Electrophoretic mobility shift assay (EMSA) is a common affinity electrophoresis technique to investigate DNA–protein or RNA–protein interactions. The rate of immigration reduction in the sodium dodecyl sulfate-polyacrylamide gel electrophoresis (SDS-PAGE) represents a protein or protein complex that is capable of binding a given DNA or RNA sequence, and stoichiometry between binding protein and DNA or RNA. It is widely used in determination and quantification of interaction between ribosomal transcription factors and promoter sequences with SNPs or in some cases between translation factors and 5' UTR.

To take into account epigenetic factors, chromatin immunoprecipitation (ChIP) was developed to reveal the binding protein within a specific genomic region or the associated genomic regions to a specific protein in the cell. After shearing the DNA or RNA into fragments, the protein-DNA or protein-RNA complexes are isolated and concentrated by using appropriate antibodies or complementary nucleotide sequences from cellular lysates. In vivo, gene activation or inactivation is regulated by epigenetic mechanisms, such as genomic DNA methylation, histone modification and post-translational modification of transcription factors. As a cell-base assay, ChIP retains the epigenetic status of the native state within a cell. Sequencing or mass spectrometry is used to analyze purified products. Complementing the more specific EMSA, ChIP is applied to whole genome analysis by combination with microarray technology or NGS, and provides an option to perform high-throughput or *de novo* analysis.

The Dual-luciferase Reporter Assay System (DLR) [205] is used to measure two luciferase activities, *Renilla* and firefly luciferase, in a single protein extract. It is a robust, simple, reproducible and highly sensitive method for examining gene expression in bacterial, yeast [206] and mammalian cells [205]. The gene encoding firefly luciferase is fused to the test promoter and integrated into the genome to be examined. Meanwhile the *Renilla* luciferase is used as a control for normalizing the assay by fusing to a constitutive promoter and also integrated into genome. The ratio of the luciferase activities represents the level of gene expression. Since both luciferase activities have a linear range covering at least five orders of magnitude, the use of DLR for rapid examination and quantification of gene expression has gained increased popularity. The only limitation for DLR is the appropriate vectors containing the luciferase reporters for different systems. However, with more organisms being adapted for the DLR assay, researchers have recently constructed a 3' UTR and specific miRNA fragment downstream of the luciferase gene to study functional SNPs located in 3' UTR [207].

Humans are a sexually dimorphic species. To ensure equivalent gene expression levels from the X-chromosome, dosage is compensated by X-chromosome inactivation [208]. For autosomes, the epigenetic phenomenon such as the expression of a certain gene is inherited in a parent-of-origin specific manner is called gene imprinting [209]. Based on allele variant expression in heterozygotes, researchers are able to examine the effect of a single SNP on gene expression [210]. The commonly used methods have been discussed in traditional SNP detection methods above. 

*In vitro* functional studies of the genes having nsSNPs are important to gain better understanding of the molecular mechanism involved in normal and mutant gene function. Most *in vitro* protein studies are started from isolated proteins from a number of sources, for example expression abundant tissue, *in vitro* expression or recombinant expression, followed by an examination of the protein carrying out its functions in a controlled environment. Here we focus on specific applications for the study of nsSNPs. Three major profiles of certain proteins are considered and investigated by *in vitro* methods; they are gene expression, protein structure and enzyme kinetics.

Protein immunization is the most commonly used technology to detect the loci, expression level, truncation and post-translational modification of proteins of interest. Immune responses can be elicited against full-length proteins or synthesized peptides with either the wild-type sequence or containing a nsSNP. The specific binding between an antigen and antibody give an exclusive antibody-antigen complex. By conjugating fluorescent, luminescent, radioactive or enzymatic labels, researchers are able to evaluate immune reaction in different ways. For example, immunofluorescence, immunoblotting and enzyme-linked immunosorbent assays are generally used. Since a monoclonal antibody is made from identical immune cells derived from one unique parent cell, in contrast to polyclonal antibodies, it has the monovalent affinity to recognize even a single amino acid mutation [211]. Compared to the other methods mentioned above, immunoassays provide a visualization method able to specify the size, locus and expression level of target proteins.

Protein structure and conformational studies are used for determining the secondary to quaternary protein structures and even protein-substrate complexes. X-ray crystallography can obtain the mean positions of atoms in a crystal from electron density, which gives the most abundant information for protein structure followed by computational analysis. However, a protein placed in a non-physiological environment can occasionally lead to aberrant conformational changes. Also, nsSNPs may change the structure of the protein and the mutant may fail to crystallize. To circumvent these problems, researchers have used nuclear magnetic resonance spectroscopy (NMR) [212] or circular dichroism (CD) spectrum [211,213]. These methods measure unstained or fixed samples in their native environment in real time. The signal from the CD spectrum reflects the protein conformation, particularly secondary protein structure. The principle is based on three hypotheses: (1) protein structure can be divided into homogeneous units with defined bond rotations parameters; (2) the contributions of different types of secondary structures are additive; (3) the contribution of bond rotations is limited to rotatory dispersion of the protein. A site-direct mutation caused by an nsSNP can alter both the secondary structural element and the bond rotations and can be evaluated by CD spectrum, by comparison with fully functional wild-type protein.

Exon sequences typically have lower frequency of nsSNPs as compared with introns, because any changes of function in the resulting protein that lead to decreased fecundity or sterility are heavily selected against. However, a number of X-linked diseases are not caused by hereditary, but rather, spontaneous rare mutations in exon region, leading to disease [214]. These mutations can cause missense, nonsense or frame-shift mutations. In most cases, frame-shift mutations generate truncated proteins just like nonsense mutations. As mentioned, high frequency of partially translated proteins in patients usually indicates a functional depletion of the absent domain [197]. It is indeed a good starter to understand the pathology of a specific gene to a disease, however, additional information is needed to link a specific effect to a domain if more than one structural domain is deleted.

For *in vitro* studies nsSNPs are selected based on several criteria. First, nsSNPs appearing in high frequencies in patients; second, an nsSNP site which is conserved and located in a homologous region; third, the resulting mutant is potentially important to the biochemical functions or structural fold. Generally, a nsSNP may not affect the structural the entire domain, instead, it is more likely to cause local structural changes.

A CD spectrum can provide information of secondary structural changes. It is usually used in combination with temperature jump techniques for monitoring protein denaturation dynamics [201]. The intrinsic protein fluorescence measurement reflects conformational changes associated with aromatic amino acids, which is an effective tool for steady-state kinetics or binding kinetics analysis [215]. These profiles are represented by thermodynamic or chemical parameters to characterize protein stabilization and protein-substrate interactions. Data generated from these analyses also provide the basis for *in silico* modeling and dynamic simulation as described in Section 1. To evaluate the effects of nsSNPs *in vitro*, results from these analyses are determined by plotting the data or fitting the data according to chemical and kinetic equations. For example, the Michaelis–Menten equation or the Hill equation is used to determine the equilibrium dissociation constant [216] and compare pathologic mutations with wild-type protein. A dysfunctional mutation in most cases leads to local structural instability [201] or decreased binding affinity [197], and in other cases some mutations result in increased binding affinity to substrate [217] or alternative substrate binding [218], which cause decreased enzyme turnover rate or competitive inhibition, respectively.

Not all the nsSNPs alter the structural stability or binding affinity. For example, arginine finger is a conserved motif in helicases, and has postulated to play a role in adenosine triphosphate (ATP) hydrolysis and energy coupling. The defect in ATP hydrolysis activity can be measured either by chemical staining method or P^32^ radiation technique. Thus, most of mutations of conserved arginine affect ATP hydrolysis and some of them appear to be crucial for energy coupling, but none of them affect ATP and DNA-binding abilities [219]. Another exception is that the nsSNPs influence functional post-translational modification of wild-type protein by changing target sites or key flanking residues. For example, phosphorylation is one of the most common post-translational modifications, and turns many protein enzymes on and off. Phosphotryptic mapping is usually used to identify inducible phosphorylation sites that may regulate protein function by comparing phosphotryptic maps of protein phosphorylated *in vitro* by specific kinase with phosphotryptic maps obtained from original tissue. Once a kinase is identified, the wild-type protein, the wild-type protein treated with kinase and phosphorylation sites of mutant protein can be examined to reveal the role of post-translational regulation [220,221].

Overall, a protein-substrate binding assay or protein kinetics assay is also frequently used depending on the nature of the proteins. Various detection methods are available to visualize protein reactions, such as intrinsic protein fluorescence, immunolabeling, dye staining, and radiation. By obtaining enough evidence to understand the molecular pathogenesis, an *in vitro* functional rescue is supposed to be considered, since such disease caused by functional exon SNP is most likely to be targeted compared to SNP located in another region. Testing of potential pharmacological agents can lend insight into the malfunctioning protein and will determine if the protein can regain its wild-type activity *in vitro* leading to rescue of cell function [222].

## 4. *In Vivo* Analysis of Pathogenic Mutations

### 4.1. Zebrafish as a Model for Studying the Downstream Effects of Non-Synonymous Single Nucleotide Polymorphism (nsSNPs)

The canonical human disease model in fish is the zebrafish (*Danio rerio*). Practically all zebrafish genes have human equivalents with conserved molecular functions [223]. The many advantages of the zebrafish embryo and adult fish have been well documented [223,224,225,226]. SNPs have been employed in identifying the causative mutation of multiple diseases, through forward genetic screens, involving exposure of a male founder fish to a mutagenic compound, ENU (*N-*ethyl-*N-*nitrosourea), followed by screening for offspring with distinctive phenotypes; morphological, behavioral and molecular. Known SNPs that are found to associate with the phenotype using linkage studies have enabled positional mapping and genome wide association studies to identify the causative mutations [227]. Conversely, reverse genetics has mostly involved knockdown or knockout of genes of interest, followed by studies of the mechanism and resulting phenotype. The next step in this evolution is to systematically mutagenize single nsSNPs within a gene, and study the molecular mechanism and resulting phenotype. The benefits of using zebrafish are several fold; multiple lines of fish can be created simultaneously, fish take up little room and are easy to house, there is a relatively short three month generation time for zebrafish—complemented by the number of embryos that can be produced by the founder fish—the large amounts of material available will expedite simultaneous morphological, behavioral, and molecular genetic analysis.

The zebrafish provides a uniquely accessible organism with regard to genetic manipulation, as gene editing tools have sufficiently progressed to efficiently and reliably mutate single base pairs *in vivo* (discussed below). The heritably of such a mutagenesis strategy allows for the creation of lines of fish, each carrying a target nsSNP, which can be crossbred to fish carrying markers that will assist in analysis. However, this approach has some caveats. The goal is to mutagenize a single base pair *in vivo*, rather than produce a knockout or knockin of an entire gene. This means that the numerous conditional transgenic techniques that are available in zebrafish to spatially and temporally control expression [228], may not be relevant.

Additionally, in case of X-linked genes, the effect is gender specific and it is not known how long males will survive. Moreover, the phenotype in heterozygous females is determined in part by X-inactivation, an apparently random process. It is likely that nsSNPs in zebrafish embryos may exhibit the same phenotypic outcomes. It remains to be determined if hemizygous females are sufficiently healthy to breed. Thus, one may have to resort to repeated mutagenesis for each batch of embryos, to produce sufficient material required for analysis. In this case, rather than breed mutagenized lines to zebrafish carrying reporter genes, one may need to perform the mutagenesis in embryos originating from several reporter gene lines. One of the great strengths of the zebrafish model is its adaptability in meeting new challenges. Developing a successful strategy for gene editing will allow to determine the downstream effects of nsSNPs, within the whole organism, individual tissues and down to the single cell level.

### 4.2. Gene Editing to Introduce Individual nsSNPs within the Genome

Several gene editing tools are now readily available; zinc-finger nucleases (ZFNs) [227], transcription activator-like effector nucleases (TALENs) [229,230,231,232] and the RNA guided CRISPR (clustered, regularly interspaced, short palindromic repeats)-Cas9 nuclease (CRISPR-associated enzyme) system [183,232,233]. These tools have become invaluable in manipulation of DNA in cell lines and *in vivo*, for example, in transiently transfected zebrafish embryos, or to establish heritable zebrafish lines.

Recently, two research groups independently capitalized on the CRISPR-Cas9 system to perform genome scale knockouts of almost every gene in human cells [184,234]. Thus, a lentiviral expression vector with sgRNA (single-guide RNA), Cas9 and a puromycin selection marker, called lentiCRISPR [234] were developed. With 3–4 sgRNA’s per gene, a library with over 18,000 genes was developed, targeting 5' constitutive exons. It is, therefore, easy to appreciate why the CRISPR-Cas9 system has quickly become the method of choice, when such high throughput screens are possible. Moreover, the system requires on just two components. Firstly, CRISPR normally functions as a type of acquired immune defense system that protects eubacteria and archaea cells from viruses, plasmids and phages [235,236]. CRISPR is an RNA molecule that acts as a guide RNA (gRNA), and when directly introduced into cells together with the second component, the Cas9 enzyme, which can induce double stranded cuts in DNA, effectively edits the DNA. Cas9 is a nuclease derived from a species of streptococcal bacteria and is recruited to the target DNA by the gRNA, where it cuts the DNA inside the gene of interest [237].

Several design elements should be incorporated into the system: the gRNA should be ~80 nucleotides long, consisting of two regions: 20 nucleotides at the 5' end of the gRNA that are complementary to and bind the target DNA, the remaining nucleotides are designed to form a hairpin structure with variable length, depending on the plasmid selected to expresses the gRNA. The role of the hairpin is unclear, but may help to orient the gRNA for DNA binding and aid in forming a complex with Cas9. Additionally, the 5' end of the gRNA must bind to DNA with the sequence –NCC, *i.e.*, any base pair, indicated by N, immediately adjacent to two cytosine residues. The gRNAs appear to bind most readily to DNA where the opposite strand contains a –NGG sequence, also known as PAM (protospacer adjacent motif).

Multiple versions of Cas9 containing plasmids are available from the plasmid repository at Addgene (http://www.addgene.org/CRISPR) and several software programs are available to identify 20-base-pair regions in a DNA region of interest, including Massachusetts Institude of Tecnology’s CRISPR Design (http://www.crispr.mit.edu), the German Cancer Research Center E-CRISP (http://www.e-crisp.org/E-CRISP/designcrispr.html) and the ZiFiT targeter (zifit.partners.org/ZiFiT). The first two also scan the whole genome to identify sequence regions of gRNA which might bind to similar, off-target sequences.

Once the gRNA and Cas9 enzyme are expressed in cells, the complex does the rest and cuts both strands of the target DNA. The efficiency of gene editing needs to be tested, in addition to sequencing the DNA, to directly assess if the intended DNA was correctly targeted. Strategies for reducing off-target effects include transfecting the lowest amounts of Cas9 and gRNA expression plasmids that are necessary for on-target activity, or using a mutant version of Cas9, called Cas9 nickase, which cuts only the strand of DNA that binds the gRNA. Expressing Cas9 nickase in cells with a pair of gRNAs that bind different strands of the same DNA target results in double stranded nicks whose repair then leads to mutations.

For certain applications ZFNs, which recognize longer stretches of target DNA and TALENs, which are fusions of a nuclease enzyme and DNA-binding domain protein, may provide alternative approaches [238,239]. If using the ZFN and TALEN systems, generally about a dozen different TALENs and many more ZFNs have to be tested, with DNA-binding domains that recognize different target sites, to find ones that work [240]. Moreover, the TALEN complex is less reliable at avoiding off-target sites. A much older method called site-directed mutagenesis, available as a PCR kit has several other considerations [241]. The PCR reaction does not maintain the original methylation of the DNA of interest, mixed template and reaction products have to be separated, the edited DNA requires the use of a retroviral vector for insertion into the genome and the insertion sites are difficult to control. Moreover, in very large genes the PCR method may be unsuitable due to the necessity for targeted primers, which work best at the 5' and 3' ends of the DNA. Manipulating sites outside of the 5' and 3' ends might mean having to perform PCR on a fragment of the DNA and then undertaking the extra step of DNA splicing in order to achieve the correct construct.

## 5. Case Study: *KDM5C* and *MECP2* Genes

**Figure 4:**
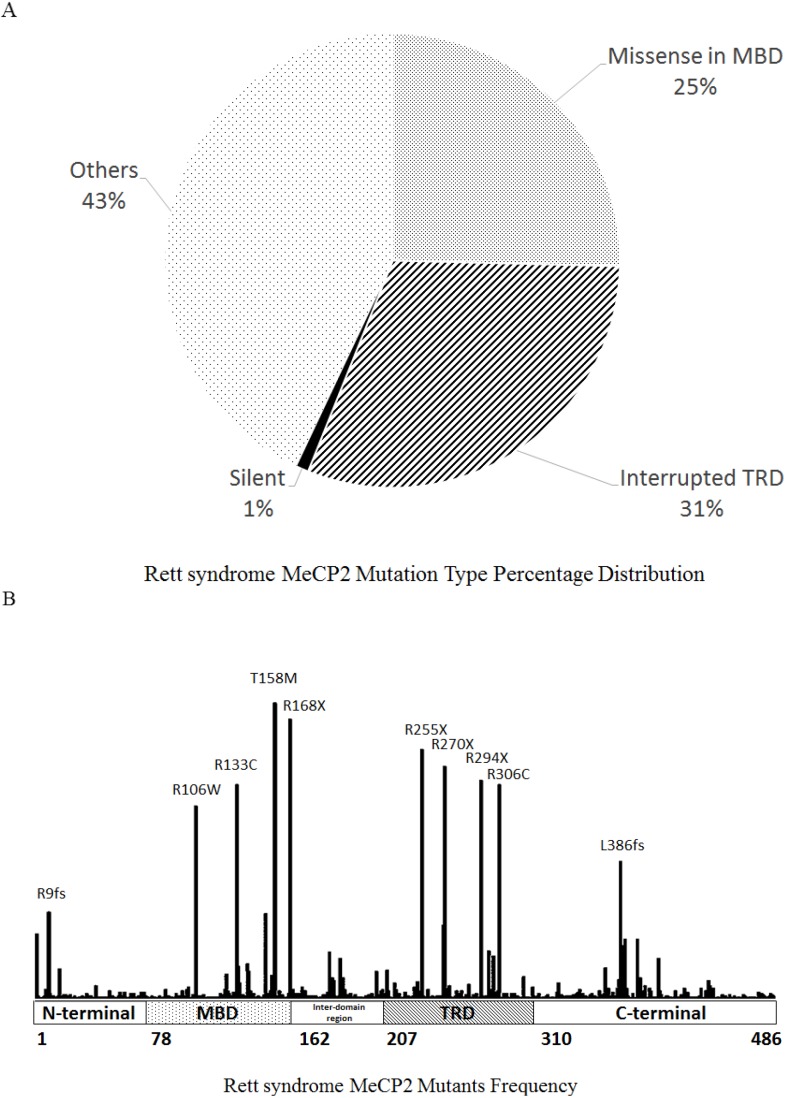
(**A**) Percentage distribution of different Rett syndrome mutants. Three major types of sequence changes in specific domain are shown in the pie chart. Missense mutants in the Methyl-CpG-binding domain (MBD) and nonsense mutations that interrupt the transcription repression domain (TRD) are the major types. Synonymous mutations in exons and all mutants in the 5' UTR, the 3' UTR and introns are termed Silent; (**B**) The location and frequency of *MECP2* Rett syndrome mutants. The most frequent 10 mutants are labeled. R106W, arginine to tryptophan point mutation at residue 106; R133C, arginine to cysteine point mutation at residue 133; T158M, threonine to methionine point mutation at residue 158; R168X, arginine to stop codon at residue 168; R255X, arginine to stop codon at residue 255; R294X, arginine to stop codon at residue 294; R306C, arginine to cysteine point mutation at residue 306; R9fs, frame-shift from arginine at residue 9; frame-shift from lysine at residue 386. The first eight mutants are termed mutational “hotspots”. Data from the *IRSF* *MECP2* Gene Variation Database.

Since the first discovery of *MECP2* [194] and *KDM5C* [242] as X-linked genes that contribute to Rett Syndrome and Mental Retardation, X-linked, Syndromic, Claes-Jensen type (MRXSCJ) [196,243,244], further disease-causing mutations have been identified [245,246]. However, the progress of functional studies on both gene products is quite different, because of different availability of corresponding protein. For *KDM5C*, only limited functional studies on domains from homologue genes are available, and most studies are still confined to genetics. On the other hand, even before the Zoghbi group found the mutations in *MECP2* causing Rett syndrome [196], the ability to bind specifically to methylated DNA and the transcription repression capabilities of *MECP2* were well understood [172,195,247,248], which provided a good basis for further investigating the effects of nsSNPs. Zoghbi’s group reported three missense mutations in the region encoding the highly conserved methyl-binding domain (MBD), and a frame-shift as well as a nonsense mutation that interrupts the transcription repression domain (TRD), by directly sequencing PCR product containing the coding exons and portions of the 3' UTR from patients. To date, *IRSF*, the *MECP2* Gene Variation Database (http://mecp2.chw.edu.au/) has 3271 mutations listed, which have been identified from Rett syndrome patients. The mutations are categorized into two main types, missense in the MBD and interrupted in the TRD shown in Figure 4A, with 43% being other mutations and 1% silent. The frequency for mutations encoded by the exon are shown in Figure 4B. Based on these data, biochemists are able to perform functional studies to reveal the effect of nsSNPs resulting in Rett syndrome.

The first study on the functional consequences of human *MECP2* mutations causing Rett syndrome was reported only one year after the SNP screening results [197]. Eight of the most frequent mutations found in patients are termed mutational “hotspots” (Figure 4B). Eleven mutations, including four missense mutations R106W, R133C, F155S, T158M and seven nonsense mutations L138X, R168X, E235X, R255X, R270X, V288X, R294X, were investigated for their effects on protein stability, methyl-CpG-binding and transcriptional repression capabilities [172,195,247,248]. Mutations found within the MBD abolished methylation specific binding to the DNA substrate and target genes lost the repression imposed by MeCP2 protein. The mutations causing the interruption of the TRD domain led to a failure to recruit the transcriptional repressors Sin3A and HDAC1 [249]. Further studies of the Rett syndrome mutants indicated that the MeCP2 protein can 1) bind tightly to biochemically defined 12-mer nucleosome arrays [199]; (2) bind to four-way junction DNA in a structure-specific methyl-CpG-independent manner [200]; (3) compact chromatin via its three AT-hook domain [204]; and (4) bind specifically to 5-hydroxymethylytosine, enriched in the target gene promoter [202]. Biophysicists have performed structural studies using CD spectrum, inner fluorescence [201] and crystallography [144] and these data indicate that the Rett syndrome mutants have a unique role in DNA binding causing reduced thermal stability and an increased binding to methyl-CpG DNA. Through different methodologies used in these studies, one has a better understanding of the molecular mechanisms leading to Rett syndrome. However, the relationship between the nsSNPs in MeCP2 protein and pathogenesis are still unknown.

A breakthrough was made by Greenberg’s group in 2003 [198], where they used an EMSA to show that the MeCP2 protein bound to the *BDNF* gene promoter III (later corrected to promoter IV) is a potent modulator in many aspects of neuronal development, and regulates the transcription of the *BNDF* gene. This process is further regulated by KCl induced membrane depolarization that triggers calcium-dependent phosphorylation of MeCP2 protein. Recent studies performed by Chromatin Immunoprecipitation followed by ChIP-Seq have identified even more downstream targets with altered gene expression in *MECP2* mutant mice [250,251]. These results demonstrate that *MECP2* has multiple roles in brain development and point the way for further pathogenesis studies [130].

Recently, four new activity-dependent phosphorylation sites (S86, S274, T308 and S421) were revealed, that might regulate the *MECP2* function using phosphotryptic-mapping techniques [221]. This study identified phosphorylation of T308 as an activity sensor that controls the interaction of MeCP2 protein with nuclear receptor co-repressor (NCoR/SMRT) complex. At the same time, it was also found that the mutational hotspot R306C, proximal to the T308 phosphorylation site, abolishes the binding to the NCoR complex [203]. These two studies implied that the two mutations have similar contributions to the neurological deficits in Rett Syndrome. Methods used in *MECP2* studies along with the historic timeline of research milestone are listed below (Table 2).

Using the above methodology, an *in silico* analysis of the nsSNP of interest was performed as a proof of principle. Several web servers were utilized to predict *in silico* the stability changes upon mutations; D87G, and D402Y in the KDM5C protein, and the mutations; D97Y, L100R, R106W, R111G, Y120P, Q128P, E137G, P152R and F155S in the MeCP2 protein (Table 3). The structure of human KDM5C, which is a large protein with 1560 residues, is not available, thus, the structure was built through homology modeling using JACKAL [252,253]. First, sequence alignments were done using Psi Basic Local Alignment Search Tool (PSI-BLAST) in National Center for Biotechnology Information (NCBI) to identify conserved regions and identical structures. The D87G mutation in KDM5C is in an AT-rich interaction domain (ARID) and the sequence of this domain and the protein with PDB ID 2JRZ were found to have a 98% identity with no gaps in sequences. Therefore, this structure was used as a template in homology modeling. The region containing the D402Y mutation was modeled using two different structures. One of them is the protein with PDB ID 3DXT, which is 38% identical with 11% gaps, and the other one is the protein with PDB ID 4IGO, which is 54% identical with 6% gaps. Since there are no experimental results to compare to, both of these structures were independently utilized as a measure of consistency in results.

**Table 3:** Webservers used in the case study along with short description of their functionality.

Webservers	Input	Method Summary
MuStab	Sequence	Support vector machine (SVM), trained on various amino acid features
dFIRE/DFIRE2	Structure	Statistical potentials with orientation-dependent interactions
FoldX	Structure	Potential with weighted sum of empirical contributions to free energy
Eris	Structure	Molecular mechanics type potential with weighted sum of contributions to free energy

Two structures were used for MeCP2 protein modeling, Protein Data Bank (PBD) ID 1QK9 (the human MECP2 structure) and PBD ID 3C2I, which has 94% identity with no gaps in the sequence. After building the structures using JACKAL, the termini were patched and hydrogen atoms were added using Visual Molecular Dynamics (VMD) [254]. The wild-type structures were (1) relaxed for 500 steps with backbone atoms restrained with 5 kcal/mol-Å force constant; (2) the proteins were energy minimized for another 35,000 steps with no restrains ensuring full convergence; (3) the mutant proteins were built using these structures in JACKAL; and (4) the mutants were minimized for 500 steps using Nanoscale Moleclar Dynamics (NAMD) [255,256].

After that, the MuStab [21,22] server was utilized, which uses a sequence-feature based prediction with support vector machines. The parts of the sequences used to prepare the wild-type structures were fed into the server together with the pH and temperature information from experimental structures. The server identified the mutants as stabilizing or destabilizing with corresponding confidence values of the predictions. Then, the wild-type and mutant structures described above were used in the (dipolar DFIRE (dDFIRE) and DFIRE2 [35,36] server, which uses a structure-based statistical potential, and subsequently the dDFIRE and DFIRE2 energies were obtained for each structure. The stability changes upon mutations were calculated by subtracting the wild-type energies from mutant energies. Finally, the Eris [124] and FoldX [33,34] servers were utilized, which make structure-based predictions of stability changes upon mutation based on first-principles. The same wild-type structures were used in these servers and the servers perform the mutations and use their mutant structures to calculate the ∆∆*G* upon the mutations. In summary, except for the MuStab servers, the other three servers produced positive or negative ∆∆*G* values indicating destabilizing or stabilizing mutations.

In summary, all four servers produced consistent results in general for nine mutants out of 11 as summarized in Table 4. The consistency of the results was assured in three aspects. First, the results were collected from several servers, which utilize different methodologies; Second, different structures were used for the same regions of the proteins where applicable and the results from those were collected independently and compared; Third, the Eris and FoldX results were collected from a reverse path. For example for the D87G mutant of KDM5C protein, the mutant structure was utilized and the servers were used to mutate it back to the wild-type to determine whether an opposite effect was obtained or not. An opposite effect was seen for this mutant from both Eris and FoldX. This was specifically done to improve the initially inconsistent results of the two MECP2 mutants (D97Y and L100R) shown in Table 2. In summary, two KDM5C mutations, D87G and D402Y, were successfully identified as destabilizing and stabilizing, respectively. Also, eight MECP2 mutants, namely L100R, R106W, R111G, Y120P, Q128P, E137G, P152R and F155S were successfully identified as destabilizing. However, in-depth studies are being performed to assess the accuracy of different web servers.

*MECP2* and *KDM5C* have multiple roles in embryonic development and proper organism function. GO annotation indicates that *MECP2* is involved in 42 biological processes (UniProtKB/Swiss-Prot: P51608), whereas five have been identified for *KDM5C* (UniProtKB/Swiss-Prot: P41229). This is an indication that the role of *KDM5C* is more restricted than for *MECP2*. Large numbers of zebrafish embryos can simultaneously undergo gene editing and then they can be non-invasively screened for morphology and behavior during early development. Analyzing the gross morphology and behavioral phenotype of mutagenized embryos goes hand in hand and changes in behavior are likely reflected in morphological abnormalities. In addition to standard gross morphological observations, hundreds of strains of zebrafish are now available with fluorescent markers driven by promoters for practically every cell type, allowing for direct, real time analysis of fluorescence in living embryos (http://www.zfin.org) [257]. Performing gene editing in transgenic reporter lines selected for neuronal and other cell types, can be scaled as needed.

**Table 4:** Stability changes upon mutations. Stabilizing mutations are highlighted in blue, and the rest are identified as destabilizing.

MECP2 Mutants	∆∆*G* (kcal/mol)								Effect, Confidence
	dDFIRE (1qk9)	DFIRE2 (1qk9)	dDFIRE (3c2i)	DFIRE (3c2i)	Eris (1qk9)	Eris (3c2i)	FoldX (1qk9)	FoldX (3c2i)	MuStab
D97Y	−1.76	−2.36	−0.73	−1.36	>10	−2.08	5.28	1.28	Stabilizing, 26%
L100R	5.95	3.89	3.66	2.10	2.68	3.93	2.3	2.13	Destabilizing, 90%
R106W	−1.47	−2.24	−0.63	−2.13	>10	9.71	10.9	4.12	Stabilizing, 27%
R111G	1.73	1.01	2.15	1.51	3.22	8.57	1.56	1.71	Destabilizing, 94%
Y120P	2.69	2.30	3.66	2.45	−8.67	7.48	0.53	1.95	Destabilizing, 90%
Q128P	1.59	0.52	0.65	0.57	>10	8	1.34	0.12	Destabilizing, 92%
E137G	2.68	1.19	3.13	1.63	3.86	5.41	2.22	2.29	Destabilizing, 88%
P152R	7.54	3.74	2.05	1.13	>10	0.44	2.28	1.95	Destabilizing, 81%
F155S	5.16	4.38	6.62	5.17	6.8	5.04	5.73	4.03	Destabilizing, 93%
**KDM5C Mutant**	**dDFIRE (2jrz)**		**DFIRE2 (2jrz)**		**Eris (2jrz)**		**FoldX (2jrz)**		**MuStab**
D87G	0.34		0.994		4.48		0.53		Destabilizing, 94%
**KDM5C Mutant**	**dDFIRE (3dxt)**	**DFIRE2 (3dxt)**	**dDFIRE (4igo)**	**DFIRE2 (4igo)**	**Eris (3dxt)**	**Eris (4igo)**	**FoldX (3dxt)**	**FoldX (4igo)**	**MuStab**
D402Y	−0.27	−1.379	0.19	−0.741	−4.14	−1.49	−1.64	−0.6	Stabilizing, 27%

As one study indicated that a point mutant of *MECP2* affects swimming behavior in zebrafish [258], assessing the highly stereotyped motor behaviors of developing embryos is targeted [259]. Within the first 24 h, embryos coil rhythmically within the chorion, as slow-muscle begins to function—this behavior can be captured using video surveillance of embryos for rapid review. Following escape, or removal from the chorion at two days post fertilization (2 dpf), embryos will display an escape response to light tactile stimulation. Thereafter, by 3 dpf the fish can swim using voluntary muscles. Velocity and turn frequency are two measurements that can be assessed using Noldus DanioVision or ViewPoint Zebrafish, two software programs commercially available. Indeed, there is a plethora of imaging strategies, including high throughput automated systems driven by numerous available software applications that can be employed to analyze behavioral phenotypes [259].

As *MECP2*, for example, results in several separate phenotypes we expect that these analyses will identify characteristic spatial and temporal differences for each phenotype. Additionally, by using video tracking of development and fluorescent reporter lines, subtle differences for individual nsSNPs within a phenotype can be identified. Rett syndrome has at least three subtypes, but the spatial and temporal molecular mechanisms are virtually unknown. The subtle, or possibly not so subtle, differences in downstream effects leading to the clinically observed phenotypes are essentially a mystery. The data from these preliminary, non-invasive observations can provide insight into the mechanism and progression of disease phenotypes caused by individual nsSNPs. Additionally, critical developmental time points, such as the onset of fluorescent reporter gene changes can be identified and inform molecular genetic analysis that can be performed simultaneously.

One of the major objectives in the field is to understand the disease mechanism. To achieve this, the spatial and temporal gene expression changes, which are expected in embryos when pathogenic nsSNPs are present, need characterizing. Indeed, even the normal downstream interactions of *MECP2* and *KDM5C* are not yet fully established. Molecular genetic analysis can include genomics, transcriptomics (gene expression arrays), and proteomics. The purpose is to determine the omic signature for every nsSNP within a region of interest, whole gene, or through high throughput methods, the whole genome. Using genomics the core and differential-omic signatures of each nsSNP within genes such as *MECP2* and *KDM5C* can be determined. As already discussed, several diseases arise from multiple nsSNPs within the multiple binding domain (MBD) of MeCP2 protein. The MBD encompasses a 222 bp/74 amino acid region and within this a DNA binding domain a 90 bp/30 amino acid region is identified. A key question is if there is a core -omic signature that can be identified linking multiple nsSNPs to a single disease state and if there are distinct differential signatures between groups of nsSNPs.

Evaluating the transcriptome of mutagenized embryos utilizing NGS is an important step in understanding the molecular basis for each phenotype. To facilitate a comprehensive or more focused analysis, such as in neuronal tissue in X-linked mental retardations, analysis of the transcriptome at critical developmental stages, identified during morphological and behavioral analysis, can be performed using a variety of samples, including whole embryos, selected tissues, multiple pooled embryos, single specimens and single cells. The objective of this approach is to establish the core genetic markers, both unregulated and downregulated genes, for each nsSNP. Depending on the desired information the analysis can focus on the earliest effects of a nsSNP prior to the overt appearance of the disease, the core changes in gene information at the point of onset of morphological anomalies, the appearance of behavioral symptoms, or even when the disease is established and clinical symptoms are present. These data can facilitate comparative genomics between each mutation, for example, the two KDM5C mutations, D87G and D402Y, one destabilizing and the latter stabilizing. In MECP2 mutants, nsSNPs that are all destabilizing, are namely L100R, R106W, R111G, Y120P, Q128P, E137G, P152R and F155S. In the case of both *MECP2* and *KDM5C* genes, which both display polyphenotypic outcomes, identifying the core and differential genetic changes for and between each nsSNP tested can facilitate development of a genetic fingerprint database.

Using the transcriptome screening method, all pathogenic nsSNPs in a gene can be evaluated, lending insight into the molecular mechanism governing each phenotype. Comparative genomics will confirm: (1) the nsSNPs within a single gene that display equivalent transcriptomes; (2) separate out nsSNPs with differential RNA signatures, even though they are classified as causing the same disease; and thus (3) establish grouping of nsSNPs based on their molecular signature, which will lend itself to analysis of the underlying mechanism. Based on clinical studies, it can be predicted that all nsSNPs causing Rett syndrome, for instance, will share a core RNA signature, and that the pleiotropic effect of nsSNPs within a single gene, will have a transcriptome correlated to the disease outcome. It is tempting to speculate that all nsSNPs in the DNA binding domain of MECP2 would result in Rett syndrome. Clinical data, however, refute this notion, with individual DNA binding domain nsSNPs not only causing phenotypic variations within Rett syndrome itself, but also entirely different disease phenotypes such as Encephalopathy (neonatal severe). As an illustration, this methodology has the potential to reveal the causative differences between the transcriptome of three Rett syndrome subtypes, including classical Rett syndrome [196], Rett syndrome with preserved speech variant [260] and Rett syndrome with the Zappella variant [261,262], and also identify the causative differences between each of the several disease specific phenotypes.

To accelerate analysis, and develop a readout for potential therapeutics, establishing an RNA signature for each nsSNP or group of SNPs, if they prove to have a collective RNA signature, is needed. Using a digital, molecular barcoding chemistry (Nanostring Technologies, Seattle, WA, USA), up to 800 genes can be profiled simultaneously. Over and above that, it is now possible to develop nCounter Elements assays in lab. Moreover, the readout is quantitative, indicating transcript number for each gene tested. Once a set of unique RNA signatures is established for groups of nsSNPs exhibiting the same phenotype, it will no longer be necessary to perform whole genome transcriptomics, rather, nCounter assays can synchronously analyze multiple samples, providing a direct readout of each signature. Given the speed, reproducibility and sheer number of genes/set of signature that can be analyzed simultaneously it seems likely that this technology is set to overtake Reverse Transcription-PCR (RT-PCR) as a screening method. 

The use of an RNA signature can be expanded as a comparative tool between species, in testing the effects of morpholino knockdowns, for example, or comparing zebrafish lines carrying various genetic manipulations. A few mutant lines are currently available from Zfin. *MECP2*, (RefSeq: XM_005166687) has a mutant line available, *mecp2fh232/fh232*(*AB*) and *KDM5C* (RefSeq: NM_001123234), has four known mutants; la026535Tg, la026536Tg, sa15146 and sa17413. These can be used as proof of principle for the RNA signatures. Indeed this need not to be limited to zebrafish, but can be extended to mouse lines and even human samples, either lab generated or patient samples. 

To further evaluate the effect of key candidate downstream effectors whole mount zebrafish are amenable to high throughput, high resolution *in situ* hybridization screening using robotic processors [263,264]. This data is essential to provide a tissue and cell level analysis when investigating disease mechanisms.

Finally, zebrafish reporter lines can be established, based on the above analyses, with fluorescent marker gene expression, driven by the regulatory element of sentinel markers. This will provide transgenic zebrafish embryos to screen bioactive small molecule, biologics and other potential therapeutic interventions.

## 6. Conclusions

In the age of rapid advances of genome sequencing techniques, the development of methods for predicting disease-causing missense mutations or nsSNPs has gained increased significance. The straightforward approach is to build a library of DNA defects associated with particular diseases, *i.e.*, database of genotypes causing a particular disease. The increasing number and size of such databases is essential for fast and precise diagnostics, since the only information required is the individual’s genome. Once the individual genome is mapped onto the database of the diseases’ genotypes, the prediction of the disease predisposal can be done instantly. However, recent completion of the 1000 genomes pilot project [265] revealed that most individuals carry 250–300 loss-of-function variants in annotated genes and 50–100 variants previously implicated in inherited disorders [266]. In addition to this observation, it is known that the severity of a disease depends on many factors, and, for individuals carrying the same disease-causing mutation(s), the manifestation can be quite different.

These observations indicate the necessity of further development of approaches to predict the disease-causing effect without the help of databases and comparison. As outlined in this review, the best approach, perhaps, is to rely on collective efforts that utilize wider perspectives from both computational and experimental approaches. It is unlikely that *in vitro* and *in vivo* approaches will be applicable for investigating each gene variation, so *in silico* methods can be applied first to deliver testable hypotheses and to reduce the number of candidate genes/variations to a number appropriate for experimental testing. *In vitro* experiments provide a direct measure of the effect of mutation(s) on the biochemical reactions associated with a particular gene and can also be very insightful in revealing the effect of mutation(s) on various biophysical characteristics as stability and interactions. Also, results from those experiments such as thermostability play a key role in the development and validation of *in silico* methods. However, such experiments are time and labor intensive and require careful selection of target genes and mutations. At the same time, the *in vitro* experiments are the ultimate proof of the biophysical effects caused by the mutations. With that being said, however, to understand the overall effect of a gene variant requires *in vivo* studies, which are more directly relevant to human disease than *in vitro* studies. For example, the neuropathology (phenotype, behavior, brain morphology and function) of variants involved in X-linked mental retardation can only be discerned in a whole organism. For initial studies, zebrafish are highly suited to this task as they are genetically tractable, available in large numbers, develop quickly and external to the mother. Importantly, they have similar organ systems to mammals, share significant genetic sequence identity to humans and in many cases, within five days of the onset of development, begin to manifest disease phenotypes. These phenotypic and genetic similarities to humans make zebrafish superior to flies, worms and yeast screens. The large number of fluorescent reporter lines available, driven by cell specific enhancers in this optically clear organism allow for live imaging directly in the whole organism. This provides a real-time readout of affected cells, tissue and organs, which is not possible in mammalian models. The zebrafish is thus extremely useful and cost efficient when screening large numbers of variants for behavioral, morphological, physiological and molecular pathway analysis, including downstream genetic and epigenetic outcomes [267,268]. Further studies can then be transferred to mice and other mammalian models as appropriate. These studies are lengthy, time consuming and expensive, but vital in the next stage of disease modeling. By narrowing down variants of interest highly focused mammalian studies are more efficient and productive. In combination, *in silico*, *in vitro* and *in vivo* methods all have particular roles in identifying and understanding the mechanism of disease variants.

## Abbreviations

MeCP2: methyl CpG binding protein 2
nsSNP: non-synonymous single nucleotide polymorphism
NGS: Next Generation Sequencing
RFLP: Restriction Fragment Length Polymorphism
SSRs: Simple Sequence Repeats
PCR: Polymerase Chain Reaction
ASPE: Allele-Specific Primer Extension
SBE: Single Base Extension
FRET: fluorescence resonance energy transfer
L-RAC: Ligation-rolling Circle Amplification
MIP: Molecular Inversion Probe
UTR: Untranslated Regions
EMSA: Electrophoretic Mobility Shift Assay
SDS-PAGE: Sodium Dodecyl Sulfate-Polyacrylamide Gel Electrophoresis
ChIP: Chromatin Immunoprecipitation
DLR: Dual-luciferase Reporter Assay System
NMR: Magnetic Resonance Spectroscopy
CD: Circular Dichroism
ATP: Adenosine Triphosphate
MBD: Methyl-CpG-binding domain
TRD: Transcription Repression Domain
ARID: AT-rich interaction domain
DNA: Deoxyribonucleic Acid
RNA: Ribonucleic acid
SVM: Support Vector Machine
GOASVM: Gene Ontology Annotation SVM
MLSTA: Machine Learning for Protein Stability
DFIRE: Distance-Scaled, Finite Ideal Gas Reference
PoPMuSiC: Prediction of Protein Mutant Stability Changes
dDFIRE: dipolar DFIRE
SNPs&GO: SNPs and Gene Ontology
CELLO: Subcellular Localization Predictor
ngLOC: n-gram-based Bayesian Localization Predictor
PPT-DB: Protein Property Prediction and Testing Database
PROFcon: Prediction of long-range Contacts
ProA-RF: Protein Aggregation Prediction Server-Random Forest
ProA-SVM: Protein Aggregation Prediction Server-Support Vector Machine
PASTA: Prediction of Amyloid Structure Aggregation
SDM: Site Directed Mutator
BONGO: Bonds on Graphs
MD: Molecular Dynamics
LIE: Linear Interaction Energy
NCBI: National Center for Biotechnology Information
BLAST: Basic Local Alignment Search Tool
PDB: Protein Databank
VMD: Visual Molecular Dynamics
NAMD: Nanoscale Molecular Dynamics
MuStab: Predicting Protein Mutant Stability Change
SIFT: Sorting Intolerant from Tolerant
MAPP: Multivariate Analysis of Protein Polymorphism
Align-GVGD: Alignment Grantham-Variation, Grantham-Deviation
